# Lateralized cerebral arterial blood flow and blood pressure adaptations to short-term head-down tilt: a 4D flow MRI study with cognitive function assessment

**DOI:** 10.1016/j.mmr.2026.100048

**Published:** 2026-06-23

**Authors:** Huan-Ran Hou, Ting-Ting Zhang, Ya-Wen Liu, Lin-Kun Cai, Yan Huang, Rui Wang, Hong-Yuan Wang, Jumatay Biekan, Peng-Gang Qiao, Zheng-Han Yang, Zhen-Chang Wang, Peng-Ling Ren, Peng-Fei Zhao

**Affiliations:** a Department of Radiology, Beijing Friendship Hospital, Capital Medical University, Beijing 100050, China; b Department of Statistics, Iowa State University, Ames, IA 50011, USA; c Circle Cardiovascular Imaging, Calgary, AB T2P 1H5, Canada

**Keywords:** Simulated microgravity, Head-down tilt (HDT), Four-dimensional flow magnetic resonance imaging (4D flow MRI), Cerebral arterial blood flow (CaBF), Total cerebral blood inflow (TCBI), Systolic blood pressure (SBP), Cognitive-motor performance

## Abstract

**Background:**

Simulated microgravity, modeled by head-down tilt (HDT), induces cephalad fluid shifts that perturb intracranial hemodynamics and may affect cognitive function. However, the temporal adaptation of cerebral arterial blood flow (CaBF), both during simulated microgravity and throughout the recovery phase, remains incompletely understood.

**Methods:**

In this study, 38 healthy male participants underwent a 7-day −6° HDT protocol followed by a 5-day recovery phase. Four-dimensional flow magnetic resonance imaging (4D flow MRI) was performed at 8 time points [baseline, HDT 12 h, HDT 1 d, HDT 3 d, HDT 7 d, recovery (R) 1 d, R 3 d, and R 5 d] to quantify CaBF and total cerebral blood inflow (TCBI) in the basilar artery (BA), left and right internal carotid arteries (ICAL and ICAR), and left and right middle cerebral arteries (MCAL and MCAR). Systemic vitals and fasting cortisol/renin were collected, and a computerized reaching task assessed reaction time (RT), movement time (MT), and peak velocity (PV). Time effects were tested with repeated-measures analysis of variance (RM ANOVA) or the Friedman test. Predictors of ≥10% TCBI decrease during HDT and ≥10% TCBI increase during the recovery phase were assessed using logistic regression, and flow–behavior associations were examined using Spearman correlation.

**Results:**

No significant vessel lumen area changes were found after post-hoc analysis, despite an overall difference observed in the MCAR (*χ*²=17.40, *P=*0.015). However, average blood flow significantly changed in the ICAL (*χ*²=34.16, *P<*0.001), MCAL (*χ*²=73.11, *P<*0.001), and MCAR (*χ*²=49.02, *P<*0.001), while BA was stable (RM ANOVA *F*=0.787, *P=*0.599) and ICAR showed no significant pairwise effects despite an overall difference (*χ*²=16.35, *P=*0.022). TCBI progressively declined during HDT and rebounded rapidly at the onset of recovery (*P<*0.001). Logistic regression identified systolic blood pressure (SBP) as an independent predictor of a ≥10% TCBI reduction during HDT [*P=*0.044, odds ratio (*OR*)=3.004, 95% confidence interval (CI) 1.028−8.777], and baseline cortisol levels predicted significant TCBI decreases from baseline to HDT 7 d (*P=*0.047, *OR*=1.306, 95% CI 1.004−1.699). Cognitive-motor testing further revealed phase-dependent changes, with RT and MT generally shortening, most consistently in the no-beep condition, while PV remained stable with beep but increased without beep. Apart from an exploratory negative correlation between TCBI rebound and cued PV (*r*=−0.360, *P=*0.031), TCBI changes were largely decoupled from behavioral outcomes.

**Conclusions:**

This study demonstrates vessel-specific, lateralized adaptation of cerebral arterial inflow during 7 days of −6° HDT and 5 days of recovery, with anterior circulation more responsive to posture-induced fluid shifts and TCBI gradually decreasing then rapidly rebounding after re-ambulation. Interindividual TCBI susceptibility reflects blood pressure and endocrine status, while cognitive-motor changes remain weakly coupled, underscoring the importance of incorporating early-recovery assessments into HDT studies to better characterize cerebrovascular readaptation after re-ambulation.

**Clinical Trials Registry:**

ChiCTR2500096128

## Background

Investigating changes in cerebral arterial blood flow (CaBF) under simulated microgravity is crucial for understanding the physiological adaptation of the human body to weightlessness [1], [2]. The −6° head-down tilt (HDT) bed rest model is a well-established ground-based analogue of simulated microgravity [3], [4], [5]. By reproducing the cephalad fluid shift observed during spaceflight, HDT provides a controlled framework for investigating cardiovascular and cerebrovascular responses to microgravity and has been widely used by the National Aeronautics and Space Administration (NASA) in short-term microgravity simulation studies [6], [7], [8].

Prolonged exposure to HDT has been demonstrated to induce alterations in cerebral perfusion, blood flow velocity, and vascular dynamics [9], [10], [11], [12]. However, the dynamic temporal trajectory of CaBF, particularly during the recovery process upon returning to an upright posture, remains insufficiently characterized [2]. A more precise characterization of these adaptive responses is imperative for comprehending cerebral vascular regulation under simulated microgravity and for evaluating its potential implications for astronaut health [13], [14], [15]. Such knowledge is particularly important for understanding the short-term adaptation patterns of CaBF during HDT and recovery and may inform the development of countermeasures [16], [17], [18].

Another unresolved issue concerns the relationship between cerebral arterial inflow during HDT and systemic physiological regulation as well as functional performance [19]. Cerebral perfusion is maintained through intricate interactions between vascular adaptation and systemic circulatory control. Simultaneous evaluation of these processes is therefore essential for improving the understanding of cerebrovascular adaptation mechanisms [20], [21]. In this context, four-dimensional flow magnetic resonance imaging (4D flow MRI) provides a powerful tool for longitudinal quantification of blood flow across multiple major intracranial arteries and for assessing total cerebral blood inflow (TCBI) at the system level [22], [23], [24].

In this study, we conducted a prospective cohort study using a 7-day −6° HDT protocol followed by a 5-day recovery phase in healthy adults. By integrating vessel-resolved 4D flow MRI with systemic hemodynamic monitoring and cognitive-motor assessments, we aimed to characterize the temporal evolution of CaBF and TCBI during simulated microgravity and subsequent re-adaptation. We hypothesized that: 1) simulated microgravity would induce vessel-specific and lateralized adaptations in CaBF, 2) systemic hemodynamic and endocrine factors would modulate the magnitude of TCBI reduction during HDT and its recovery, and 3) alterations in cerebral inflow would be partially associated with cognitive-motor performance changes. This integrated, longitudinal framework enabled the identification of cerebrovascular adaptation patterns and their physiological determinants, providing new insights into neurovascular resilience and recovery mechanisms under simulated microgravity.

## Materials and methods

### Study design

**Fig. 1:**
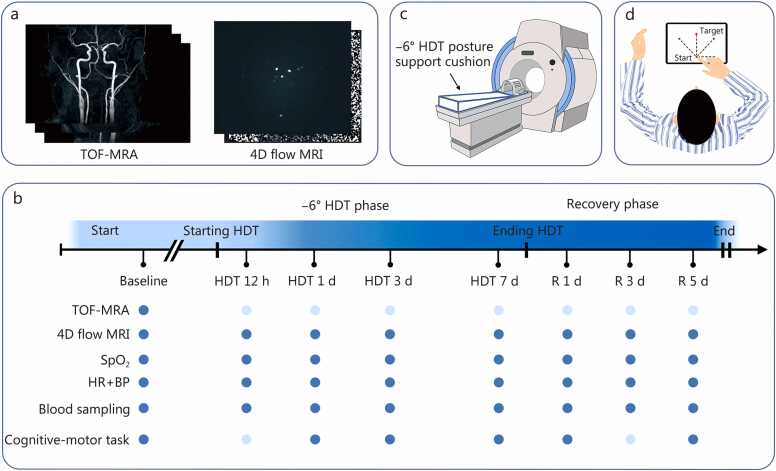
**Experimental protocol and measurement schedule during −6° HDT and recovery phases**. **a** Representative vascular images acquired during MRI, including TOF-MRA (left), which was used in the screening phase to confirm volunteer eligibility, and 4D flow MRI (right) for quantification of intracranial arterial hemodynamics. **b** Study timeline illustrating the experimental protocol, which consisted of a baseline period, a −6° HDT phase, and a recovery phase. TOF-MRA, 4D flow MRI, SpO_2_, HR, BP, blood sampling, and cognitive-motor task assessments were conducted at predefined time points (baseline, HDT 12 h, HDT 1 d, HDT 3 d, HDT 7 d, R 1 d, R 3 d, and R 5 d), as indicated by the filled circles. **c** MRI system equipped with a −6° HDT posture support cushion. **d** Illustration of the computerized cognitive-motor task. HDT. Head-down tilt; MRI. Magnetic resonance imaging; TOF-MRA. Time-of-flight magnetic resonance angiography; 4D flow MRI. Four-dimensional flow magnetic resonance imaging; SpO_2_. Peripheral oxygen saturation; HR. Heart rate; BP. Blood pressure; R. Recovery.

A total of 40 healthy male participants were recruited for the 7-day −6° HDT experiment. Participants were eligible if they were male, aged 18−58 years, 160−185 cm in height, and demonstrated normal clinical examinations and imaging findings. Specifically, fluid-attenuated inversion recovery and time-of-flight magnetic resonance angiography (TOF-MRA) were used to exclude arterial variants or abnormalities of the major intracranial arteries and to confirm normal cerebrovascular anatomy before enrollment (Fig. 1**a**). Participants were excluded if they had neurological, neuropsychiatric, intracranial organic lesions, or other serious systemic diseases; hypertension; severe allergy; malnutrition; metallic implants or other MRI contraindications. In addition, participants with 4D flow MRI data of insufficient quality for post-processing and annotation were excluded from the final analysis. All participants underwent 4D flow MRI scans, physiological measurements, and biochemical sample collection at the same predefined study time points: before HDT (baseline), 12 h into HDT (HDT 12 h), HDT day 1 (HDT 1 d), HDT day 3 (HDT 3 d), HDT day 7 (HDT 7 d), and recovery day 1 (R 1 d), day 3 (R 3 d), and day 5 (R 5 d) [25]. Cognitive-motor tasks were performed at the same time points except for HDT 12 h. The overall study timeline is shown in Fig. 1**b**. Two participants were excluded from the final analysis because their 4D flow MRI imaging was of insufficient quality for post-processing and annotation. Consequently, 38 participants were included in the final analysis.

This trial was registered in the Chinese Clinical Trial Registry (ChiCTR, No. ChiCTR2500096128). The experimental protocol was approved by the Research Ethics Committee of Beijing Friendship Hospital, Capital Medical University (2024-P2-069-04). All participants provided written informed consent before the experiment. The study adhered to the Declaration of Helsinki, and all data were anonymized during collection, processing, and analysis.

### Physiological indicator collection

Physiological indicators, including oxygen saturation (SpO_2_), heart rate (HR), systolic blood pressure (SBP), and diastolic blood pressure (DBP), were monitored to evaluate systemic hemodynamic changes during HDT and recovery [26]. SpO_2_ was measured with a portable SpO_2_ patient monitoring system (Nellcor PM10N, Covidien, Massachusetts, USA) for continuous, noninvasive monitoring of arterial SpO_2_ [27]. All physiological indicators were recorded with an electronic sphygmomanometer (Omron HBP-1300, Omron Healthcare, Kyoto, Japan) while participants maintained the experimental posture (−6° supine head-down position during HDT; seated at baseline and during recovery) to ensure methodological rigor [7]. At each time point, three consecutive measurements were obtained, and the average value was used for analysis [28]. Mean arterial pressure (MAP) and pulse pressure (PP) were calculated from the recorded blood pressure values.

### Biochemical indicator collection

Following an overnight fast of ≥8 h, blood specimens were collected from an antecubital vein at each study time point [7]. Cortisol and renin concentrations were quantified using validated chemiluminescent immunoassays in the hospital’s accredited central laboratory, following the manufacturers’ protocols [29]. Details of the assays and quality controls are provided in Additional file 1: Materials and methods.

### Four-dimensional flow magnetic resonance imaging measurements

All MRI imaging was performed on a 3.0 T Siemens Vida MRI scanner (MAGNETOM Vida, Siemens Healthineers, Erlangen, Germany) equipped with a 64-channel head and neck coil. A 4D flow MRI sequence was utilized to measure blood flow velocities. Velocity encoding (VENC) was set at 100 cm/s to optimize measurement accuracy [30]. The field of view was 229 mm × 280 mm, with an in-plane resolution of 1.59 mm × 1.59 mm and a slice thickness of 1.0 mm, providing high spatial resolution. Additional scan parameters included a repetition time of 67.3 ms, an echo time of 3.16 ms, and a flip angle of 7°. Images were acquired over 10 phases per cardiac cycle using prospective gating triggered by the peripheral pulse [30], [31]. This approach ensured precise synchronization with the cardiac cycle, enabling accurate hemodynamic assessment. To minimize circadian influences, each participant was scanned at approximately the same time of day across all measurement sessions. All participants were scanned while lying supine and flat during the baseline and recovery phases. During the HDT phase, the scanner table remained horizontal, and the −6° HDT was implemented by subject positioning using a customized cushion system placed under the body to achieve the target tilt angle (Fig. 1**c**). The scanner table and head-neck coil alignment were standardized across sessions to maintain consistent head orientation relative to the magnetic field, ensuring positional comparability between baseline and HDT scans. All participants were transported between the rest area and the MRI suite on a non-magnetic transfer bed, and the −6° posture was continuously maintained using the same cushion [25].

### Cerebral arterial blood flow analysis

**Fig. 2:**
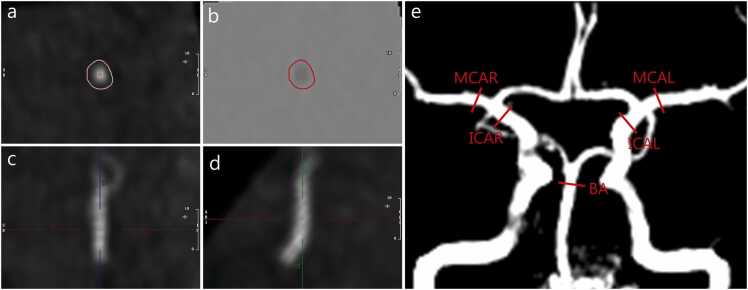
**Vascular annotation and analysis workflow using CVI 42**. **a** Cross-sectional vessel image with the ROI manually refined for precise lumen contouring. **b** Corresponding phase image showing ROI placement for velocity encoding. **c**, **d** Double-oblique reformatted views aligning the analysis plane perpendicular to the vessel axis. **e** Representative TOF-MRA showing labeling of major intracranial arteries, including BA, ICAL, ICAR, MCAL, and MCAR. TOF-MRA. Time-of-flight magnetic resonance angiography; BA. Basilar artery; ICAL. Left internal carotid artery; ICAR. Right internal carotid artery; MCAL. Left middle cerebral artery; MCAR. Right middle cerebral artery; ROI. Region of interest.

CaBF was analyzed with CVI 42 software (Circle Cardiovascular Imaging Inc., Calgary, Canada). Regions of interest (ROIs) were defined on coronal images for the major intracranial arteries (Fig. 2**a,b**) [32]. Vessel segmentation was performed automatically with manual correction when necessary, and flow parameters were quantified across the cardiac cycle using analysis planes positioned perpendicular to the vessel centerline (Fig.2**c,d**). The basilar artery (BA) and bilateral internal carotid arteries (ICAs) were selected as the principal supplying arteries to the intracranial compartment, while the bilateral middle cerebral arteries (MCAs) were used to assess distal hemispheric perfusion distribution [33]. Vascular annotations were performed at standardized anatomical locations to ensure consistency across all participants, with the annotated vessel segments shown in Fig. 2**e**. Specifically, the BA was annotated 5 mm proximal to its terminal bifurcation; the ICAs were annotated at the ophthalmic segment (C6 segment), and the MCAs were annotated 5 mm proximally to their origin [33], [34]. Detailed instructions on the data annotation process and quality control were shown in Additional file 1: Materials and methods. Intra-rater reliability of vessel annotation was assessed using intraclass correlation coefficients (ICCs), and all measured vessels showed excellent agreement (all ICCs>0.90; detailed ICC results are provided in Additional file 1**:** Table S1). Due to magnetic resonance equipment maintenance, R 3 d data were unavailable for three participants and were imputed using the median value for analysis [35], [36]. TCBI was calculated as the sum of volumetric flow rates in the bilateral ICAs and the BA [34]. Based on prior simulated microgravity literature, a 10% change threshold was used to define marked TCBI reduction (from baseline to HDT 7 d) or increase (from HDT 7 d to R 1 d) for subgroup classification and predictor modeling [25], [37].

To quantify hemisphere-specific anatomic collateral potential of circle of Willis (CoW) pathways, we computed a standardized collateral capacity score ($S_{\mathrm{CoW}}$) from high-resolution TOF-MRA. For each hemisphere, vessel diameters were normalized to the ipsilateral proximal MCA M1 segment diameter ($D_{\text{MCA}}$). The total score was defined as $S_{\text{CoW}}=S_{\text{ACom}}+S_{\text{PCom}}+S_{\text{P}1}$, where the three component scores (each 0−3) were assigned based on $R_{\text{forward}}=\min(D_{\text{A}1,\text{contra}},D_{\text{ACom}})/D_{\text{MCA}}$ for anterior cross-filling via the anterior communicating artery complex ($S_{\text{ACom}}$), $R_{\text{PCom}}=D_{\text{PCom}}/D_{\text{MCA}}$ for posterior collateral via the posterior communicating artery ($S_{\text{PCom}}$), and $R_{\text{P}1}=D_{\text{P}1}/D_{\text{MCA}}$ for the indirect posterior route indexed by PCA P1 segment caliber ($S_{\text{P}1}$). Detailed scoring thresholds are provided in Additional file 1**:** Table S2.

### Cognitive-motor task acquisition and preprocessing

Thirty-eight participants completed a rapid, goal-directed reaching task on a capacitive-touch tablet (Fig. 1**d**). The detailed task procedures and preprocessing have been described previously [38] and are provided in Additional file 1: Materials and methods. Testing was performed in a standardized posture, with participants in the −6° head-down prone position during HDT and seated at baseline and during recovery. Two participants were excluded due to anomalous data, leaving 36 participants for the cognitive-motor analyses. Reaction time (RT), movement time (MT), and peak velocity (PV) were recorded under beep and no-beep conditions.

### Statistical analysis

Data were extracted from CVI 42 using Python 3.12. Data analysis was conducted with SPSS Statistics version 27.0.0.0 (IBM Corporation, Armonk, NY, USA). Normality of the study variables was assessed using the Shapiro-Wilk test, and homogeneity of variance was evaluated for variables that met the normality assumption. Continuous variables with normal distribution were expressed as mean±standard deviation (SD), while non-normally distributed variables were expressed as median [interquartile range (IQR)]. For longitudinal comparisons across the 8 time points, repeated-measures one-way repeated-measures analysis of variance (RM ANOVA) was applied to variables meeting normality assumptions. Sphericity was assessed using Mauchly’s test. When the sphericity assumption was violated, the Greenhouse-Geisser correction was applied. The Friedman test was used for non-normally distributed variables. To further evaluate bilateral differences in paired arteries, two-way RM ANOVA was conducted for ICAs (ICAL and ICAR) and MCAs (MCAL and MCAR), with time (8 levels) and side (left and right) specified as within-subject factors. The primary term of interest was the time×side interaction, which tests whether bilateral differences vary over time. Post-hoc tests were performed only when the overall time effect was significant, using parametric tests after RM ANOVA and Wilcoxon signed-rank tests after Friedman tests. Bonferroni correction was applied across the 28 pairwise time-point comparisons for outcomes assessed at 8 time points. Binary logistic regression was conducted to identify predictors of marked changes in TCBI. To predict the ≥10% decrease from baseline to HDT 7 d and the ≥10% increase from HDT 7 d to R 1 d, HR, SBP, DBP, and MAP were entered simultaneously as independent variables. In a separate model, baseline serum cortisol and plasma renin concentrations were entered simultaneously (enter method) to test whether baseline endocrine status predicted a ≥10% reduction in TCBI from Baseline to HDT 7 d. For both logistic models, the dependent variable was coded as 1 when the ≥10% threshold was met and 0 otherwise. Results were reported as odds ratios (*OR*s) with 95% confidence intervals (CIs) and two-sided *P-*values. Spearman’s rank correlation was used to assess associations between changes in TCBI and cognitive-motor measures (RT, MT, PV) under beep and without beep conditions. All statistical significance was set at *P*<0.05 [39], [40].

## Result

### Physiological changes

The physiological indicators of 38 subjects [age: (26.7±5.7) years; height: (171.4±5.9) cm; weight: (66.9±7.2) kg; body mass index: (22.8±2.3) kg/m²] were analyzed and shown in Table 1. The $S_{\mathrm{CoW}}$ was significantly higher in the left hemisphere than in the right hemisphere (left vs. right: 3.8±1.3 vs. 3.2±1.0, *P*=0.009). The Friedman test results indicated that during the HDT and recovery phases, changes in SpO_2_ (*χ*²=8.92, *P*=0.259) and PP (*χ*²=9.53, *P*=0.217) were not statistically significant. However, HR (*χ*²=104.00, *P*<0.001) and SBP (*χ*²=23.20, *P*=0.002) exhibited significant variations. The RM ANOVA results showed that DBP (*F*=3.47, *P*=0.001) and MAP (*F*=3.90, *P*<0.001) underwent statistically significant changes during the HDT and recovery phases.

**Table 1:** Statistical analysis of physiological indicators at different scan time points during HDT and recovery (*n*=38).

**Physiological** **indicator**	**Time points**								**RM ANOVA**		**Friedman test**		
	**Baseline**	**HDT 12 h**	**HDT 1 d**	**HDT 3 d**	**HDT 7 d**	**R 1 d**	**R 3 d**	**R 5 d**	***F***	***P-*****value**	***df***	***χ***^**2**^	***P-*****value**
SpO_2_^a^ [%, median (IQR)]	98 (98−98)	98 (97−98)	98 (98−98)	98 (97−98)	98 (97−98)	98 (97−98)	98 (97−98)	98 (98−98)	-	-	7	8.92	0.259
HR^a^ [beats/min, median (IQR)]	73 (67−81)	66 (59−73)	64 (56−70)	64 (58−67)	65 (60−70)	73 (68−82)	73 (66−80)	78 (72−87)	-	-	7	104.00	<0.001
SBP^a^ [mmHg, median (IQR)]	112 (106−123)	118 (109−128)	119 (113−124)	116 (110−125)	120 (113−127)	115 (108−124)	113 (102−124)	118 (110−125)	-	-	7	23.20	0.002
DBP^b^ (mmHg, mean±SD)	66±8	68±8	70±8	71±9	72±7	70±9	68±8	70±9	3.47	0.001	-	-	-
MAP^b^ (mmHg, mean±SD)	82±8	84±8	86±8	87±8	88±6	86±9	83±6	86±8	3.90	<0.001	-	-	-
PP^a^ [mmHg, median (IQR)]	48 (42−55)	50 (43−58)	48 (44−53)	47 (42−51)	48 (43−53)	45 (40−53)	45 (38−54)	51 (40−56)	-	-	7	9.53	0.217

Values are reported as statistical results from the Friedman test or repeated-measures analysis of variance (RM ANOVA). ^a^the Friedman test; ^b^RM ANOVA. “-”. No data. SpO_2_. Oxygen saturation; HR. Heart rate; SBP. Systolic blood pressure; DBP. Diastolic blood pressure; MAP. Mean arterial pressure; PP. Pulse pressure; HDT. Head-down tilt; R. Recovery; IQR. Interquartile range

### Changes in vessel lumen area of intracranial major arteries

The vessel lumen areas of 5 major intracranial arteries were analyzed at multiple time points during the HDT and recovery phases, as shown in Table 2. The Friedman test revealed a statistically significant difference in the MCAR (*χ*²=17.40, *P*=0.015), while no significant differences were observed in the BA (*χ*²=3.59, *P*=0.826), ICAL (*χ*²=10.10, *P*=0.183), ICAR (*χ*²=9.23, *P*=0.237), or MCAL (*χ*²=11.40, *P*=0.124). Although the overall variation in MCAR was statistically significant, post-hoc analysis did not reveal any specific time points with significant pairwise differences after Bonferroni correction across the 28 time-point comparisons.

**Table 2:** Statistical analysis of vessel lumen area (mm^2^) changes in intracranial major arteries across different time points during HDT and recovery phases (*n*=38) [median (IQR)].

**Artery**	**Time points**								**Friedman test**			**Post-hoc significance**
	**Baseline**	**HDT 12 h**	**HDT 1 d**	**HDT 3 d**	**HDT 7 d**	**R 1 d**	**R 3 d**	**R 5 d**	***df***	***χ***^**2**^	***P-*****value**	
BA	22 (17−25)	20 (16−25)	22 (18−27)	23 (19−27)	20 (17−26)	21 (18−26)	21 (18−25)	20 (18−24)	7	3.59	0.826	NA
ICAL	28 (23−34)	28 (25−34)	30 (24−41)	26 (22−33)	27 (23−36)	30 (24−36)	28 (25−32)	30 (24−33)	7	10.10	0.183	NA
ICAR	29 (23−37)	29 (24−35)	30 (26−39)	28 (24−35)	28 (25−36)	31 (26−37)	29 (23−32)	29 (25−33)	7	9.23	0.237	NA
MCAL	23 (20−27)	21 (18−23)	22 (17−27)	19 (17−25)	20 (17−24)	21 (18−24)	21 (17−23)	21 (18−25)	7	11.40	0.124	NA
MCAR	22 (19−26)	24 (19−32)	24 (20−32)	21 (18−29)	22 (17−27)	24 (19−27)	22 (19−28)	21 (18−25)	7	17.40	0.015	No

The Friedman test was employed to evaluate the statistical significance of the observed variables. BA. Basilar artery; ICAL. Left internal carotid artery; ICAR. Right internal carotid artery; MCAL. Left middle cerebral artery; MCAR. Right middle cerebral artery; HDT. Head-down tilt; R. Recovery; NA. Not applicable

### Changes in blood flow of intracranial major arteries

**Fig. 3:**
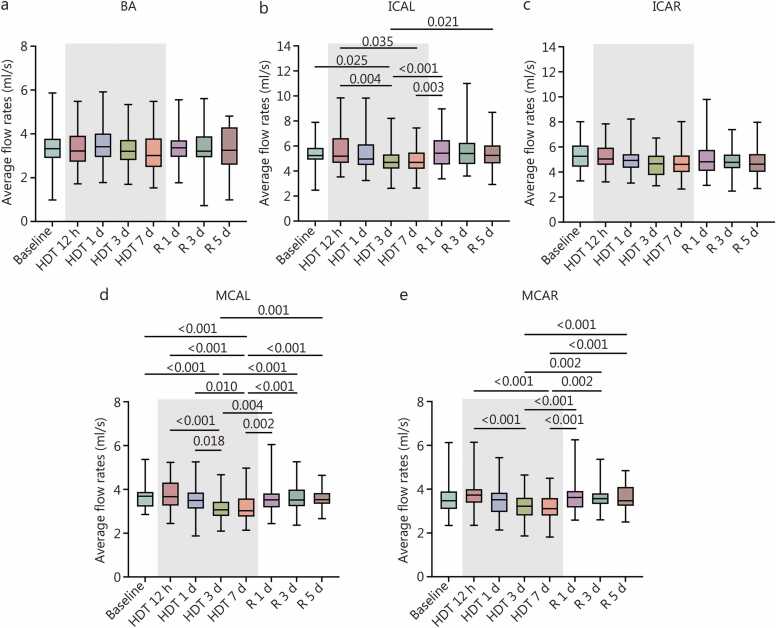
**Trends in the average flow rate of intracranial arteries across different time points during HDT and recovery phases, with significant post-hoc pairwise comparison results**. The gray-shaded region denotes the HDT phase. BA. Basilar artery; ICAL. Left internal carotid artery; ICAR. Right internal carotid artery; MCAL. Left middle cerebral artery; MCAR. Right middle cerebral artery; HDT. Head-down tilt; R. Recovery.

Fig. 3 shows the temporal trends in blood flow rates across major intracranial arteries, with significant post-hoc comparisons results. Detailed statistical analysis and indicator values are provided in Additional file 1**:** Table S3. BA remained stable across all time points, with no significant longitudinal change (Fig. 3**a**; *P*=0.599). In contrast, significant changes were observed in ICAL (Fig. 3**b**; *P*<0.001), MCAL (Fig. 3**d**; *P*<0.001), and MCAR (Fig. 3**e**; *P*<0.001), whereas ICAR showed a significant overall Friedman test result (Fig. 3**c**; *P*=0.022) but no significant pairwise difference in post-hoc analysis.

For ICAL, a decrease in blood flow was observed during HDT, with subsequent recovery at R 1 d. Significant pairwise differences were identified between the baseline and HDT 3 d (*P*=0.025), HDT 12 h and HDT 3 d (*P*=0.004), HDT 12 h and HDT 7 d (*P*=0.035), HDT 3 d and R 1 d (*P*<0.001), HDT 7 d and R 1 d (*P*=0.003), and HDT 3 d and R 5 d (*P*=0.021). In contrast, ICAR exhibited only a mild fluctuation across time points, and no post-hoc comparison reached significance.

Both MCAs showed a similar temporal pattern, with reduced flow during the middle-to-late HDT phase and recovery after HDT. For MCAL, post-hoc analysis showed that flow rates at HDT 3 d and HDT 7 d each differed significantly from multiple other time points (all *P-*values<0.05), whereas no significant difference was observed between HDT 3 d and HDT 7 d. For MCAR, significant pairwise differences were also mainly centered on HDT 3 d and HDT 7 d. Specifically, flow rates at HDT 3 d and HDT 7 d each differed significantly from other time points except baseline and HDT 1 d (all *P-*values<0.05), whereas no significant difference was observed between HDT 3 d and HDT 7 d.

**Fig. 4:**
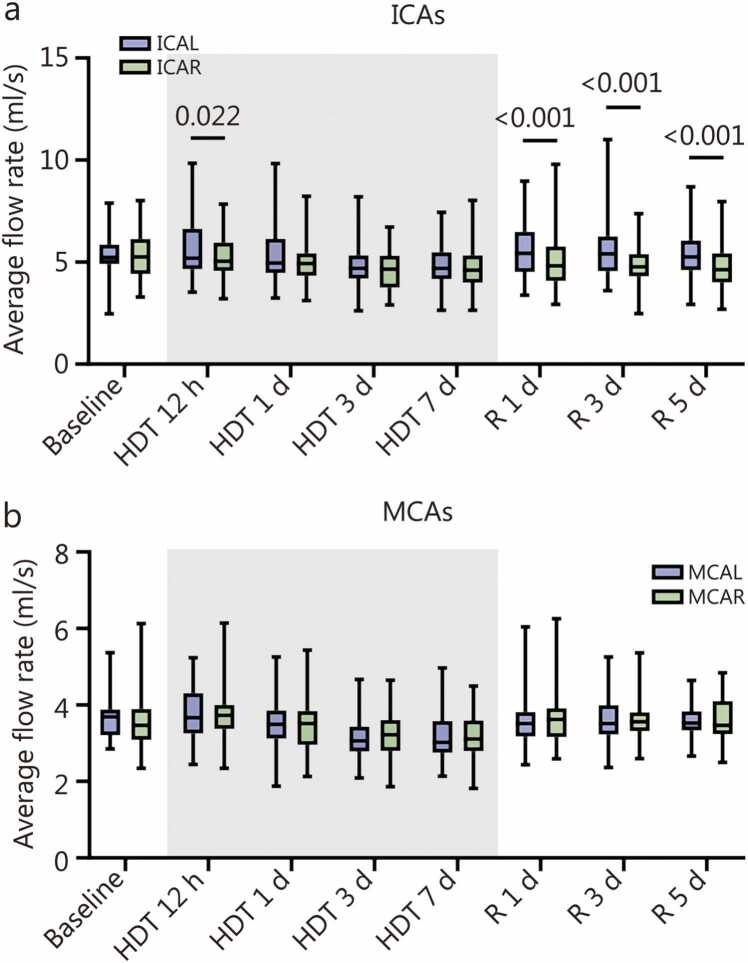
**Comparison of average flow rates between left and right paired arteries across different time points during HDT and recovery phases, with significant post-hoc pairwise comparison results for ICAs (ICAL and ICAR) (a) and MCAs (MCAL and MCAR) (b)**. The gray-shaded region denotes the HDT phase. ICAs. Internal carotid arteries; ICAL. Left internal carotid artery; ICAR. Right internal carotid artery; MCAs. Middle cerebral arteries; MCAL. Left middle cerebral artery; MCAR. Right middle cerebral artery; HDT. Head-down tilt; R. Recovery.

As shown in Table 3, the two-way RM ANOVA revealed a significant time main effect for both the ICAs and MCAs (both *P*-values<0.001). A significant time×side interaction was observed only for the ICAs (*P*=0.006), indicating that bilateral differences varied across time points, whereas no significant side main effect was detected. Post-hoc paired comparisons further localized the ICA bilateral differences to HDT 12 h (*P*=0.022) and the recovery phase (R 1 d, R 3 d, and R 5 d, all *P*-values<0.001) (Fig. 4**a**). In contrast, for the MCAs, neither the side main effect nor the time×side interaction was significant (Table 3**;** Fig. 4**b**).

**Table 3:** Statistical analysis of differences in average flow rates between bilateral ICAs and MCAs across different time points during HDT and recovery phases (*n*=38).

**Paired artery**	**Time main effect**	**Side main effect**	**Time×side interation**	**Post-hoc adjusted*****P*****-value**							
				**Baseline**	**HDT 12 h**	**HDT 1 d**	**HDT 3 d**	**HDT 7 d**	**R 1 d**	**R 3 d**	**R 5 d**
ICAs (ICAL vs. ICAR)	*F*=5.56, *P*<0.001	*F*=3.77, *P*=0.060	*F*=2.89, *P*=0.006	>0.999	0.022	0.434	0.973	>0.999	<0.001	<0.001	<0.001
MCAs (MCAL vs. MCAR)	*F*=12.60, *P*<0.001	*F*=0.02, *P*=0.897	*F*=1.60, *P*=0.135	0.050	>0.999	>0.999	>0.999	>0.999	>0.999	>0.999	>0.999

Two-way repeated-measures analysis of variance (RM ANOVA) was conducted with time and side (left and right) as within-subject factors. The table presents the time main effect, side main effect, and time×side interaction for paired ICAs and MCAs. ICAs. Internal carotid arteries; MCAs. Middle cerebral arteries; ICAL. Left internal carotid artery; ICAR. Right internal carotid artery; MCAL. Left middle cerebral artery; MCAR. Right middle cerebral artery; HDT. Head-down tilt

### Total cerebral blood inflow changes

**Fig. 5:**
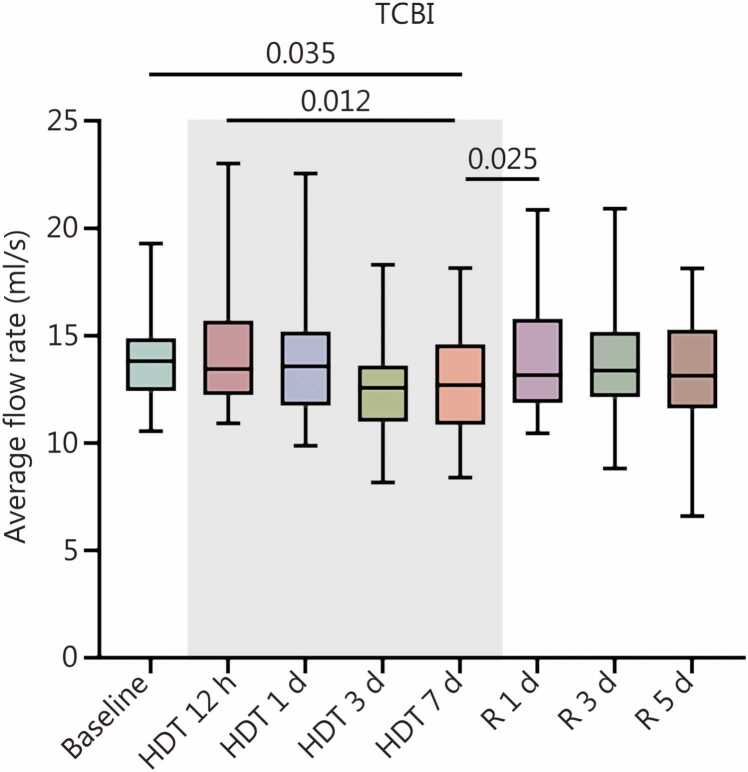
**Trends in the average flow rate of TCBI across time points during HDT and recovery**. Significance annotations are shown above brackets, and the gray-shaded region denotes the HDT phase. TCBI. Total cerebral blood inflow; HDT. Head-down tilt; R. Recovery.

The trend and statistical analysis results of TCBI are shown in Fig. 5. TCBI exhibited significant changes during the HDT and recovery phases (*P*<0.001). Before HDT 1 d, TCBI showed no significant fluctuations. Thereafter, TCBI showed a decreasing trend from HDT 1 d to HDT 3 d, followed by stabilization from HDT 3 d to HDT 7 d. Compared to baseline, TCBI was significantly reduced at HDT 7 d (*P*=0.035), and a significant decrease was also observed at HDT 7 d compared to HDT 12 h (*P*=0.012). After the end of HDT, TCBI significantly increased on R 1 d compared to HDT 7 d (*P*=0.025). Upon resumption of an upright posture, TCBI showed an increasing trend, though the change was not statistically significant.

### Total cerebral blood inflow changes and physiological indicator correlations

Binary logistic regression analysis was performed to evaluate the association between HR, SBP, DBP, and MAP with the likelihood of a TCBI reduction exceeding 10% from baseline to HDT 7 d, and a TCBI increase exceeding 10% from HDT 7 d to R 1 d. Table 4 presents the analysis of TCBI reduction by comparing HDT 7 d to baseline during the HDT phase. Among the independent variables, SBP was the only significant predictor of a TCBI reduction greater than 10% (*OR*=3.004, 95% CI 1.028−8.777), while none of the other variables showed statistical significance. As shown in Table 5, the association between the independent variables and the likelihood of a TCBI increase exceeding 10% was assessed by comparing R 1 d to HDT 7 d during the recovery phase. None of the variables demonstrated statistical significance in predicting a TCBI increase.

**Table 4:** Binary logistic regression analysis of physiological predictors for TCBI reduction between HDT 7 d and baseline.

**Variable**	**B**	***P-*****value**	***OR*****(95% CI)**
HR	0.067	0.121	1.070 (0.982−1.164)
SBP	1.100	0.044	3.004 (1.028−8.777)
DBP	2.222	0.051	9.227 (0.990−85.966)
MAP	−3.231	0.053	0.040 (0.001−1.049)

Variables entered into the model: changes in HR, SBP, DBP, and MAP from baseline to HDT 7 d. Hosmer-Lemeshow test: *χ*²=3.94, *P*=0.862; Nagelkerke *R*²=0.332. B denotes the regression coefficient, representing the change in the log-odds of TCBI reduction for a one-unit increase in the predictor. *OR*. Odds ratio; 95% CI. 95% confidence interval; HR. Heart rate; SBP. Systolic blood pressure; DBP. Diastolic blood pressure; MAP. Mean arterial pressure; TCBI. Total cerebral blood inflow; HDT. Head-down tilt

**Table 5:** Binary logistic regression analysis of physiological predictors for TCBI increase between R 1 d and HDT 7 d.

**Variable**	**B**	***P-*****value**	***OR*****(95% CI)**
HR	−0.039	0.259	0.962 (0.899−1.029)
SBP	0.050	0.885	1.052 (0.532−2.078)
DBP	0.077	0.911	1.080 (0.280−4.156)
MAP	−0.17	0.869	0.843 (0.112−6.378)

Variables entered into the model: changes in HR, SBP, DBP, and MAP from HDT 7 d to R 1 d. Hosmer-Lemeshow test: *χ*²=10.841, *P*=0.211; Nagelkerke *R*²=0.103. B denotes the regression coefficient, representing the change in the log-odds of TCBI increase for a one-unit increase in the predictor. *OR*. Odds ratio; 95% CI. 95% confidence interval; HR. Heart rate; SBP. Systolic blood pressure; DBP. Diastolic blood pressure; MAP. Mean arterial pressure; TCBI. Total cerebral blood inflow; HDT. Head-down tilt; R. Recovery

### Baseline hormonal predictors of ≥10% TCBI reduction from baseline to HDT 7 d

As shown in Table 6, binary logistic regression with baseline renin and cortisol entered simultaneously indicated that higher baseline cortisol was associated with greater odds of a ≥10% reduction in TCBI from baseline to HDT 7 d (*OR*=1.306, 95% CI 1.004−1.699), whereas baseline renin was not associated with this endpoint (*OR*=0.967, 95% CI 0.911−1.026).

**Table 6:** Binary logistic regression analysis of baseline hormonal predictors for ≥10% TCBI reduction between baseline and HDT 7 d.

**Variable**	**B**	***P-*****value**	***OR*****(95% CI)**
Renin (baseline)	−0.034	0.271	0.967 (0.911−1.026)
Cortisol (baseline)	0.267	0.047	1.306 (1.004−1.699)

Variables entered into the model: renin and cortisol at baseline. Hosmer-Lemeshow test: *χ*²=5.653, *P*=0.686; Nagelkerke *R*²=0.256. B denotes the regression coefficient, representing the change in the log-odds of TCBI reduction for a one-unit increase in the predictor. *OR*. Odds ratio; 95% CI. 95% confidence interval; TCBI. Total cerebral blood inflow; HDT. Head-down tilt

### Changes in cognitive-motor task

**Fig. 6:**
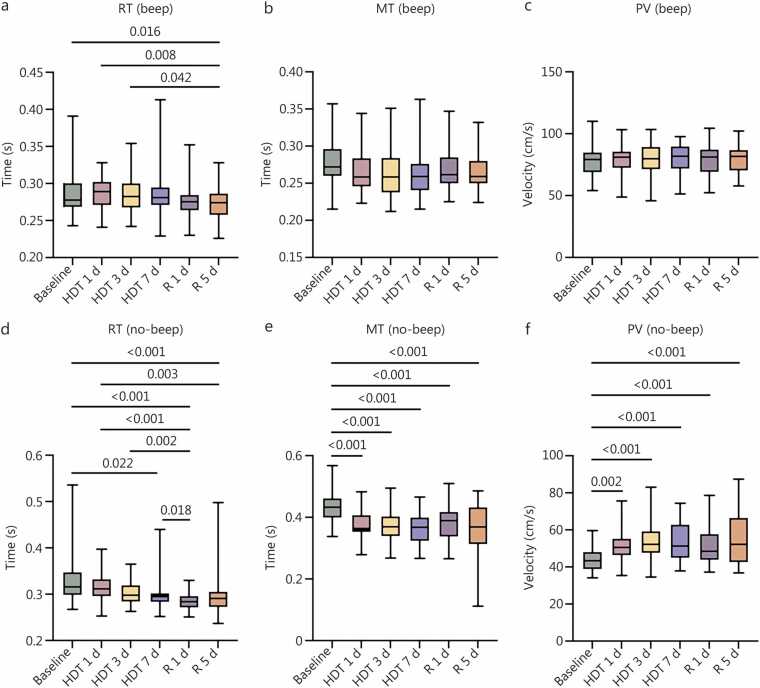
**Trends in RT, MT, and PV across time points during HDT and recovery, with significant post-hoc pairwise comparison results for RT (beep) (a), MT (beep) (b), PV (beep) (c), RT (no-beep) (d), MT (no-beep) (e), and PV (no-beep) (f)**. The gray-shaded region denotes the HDT phase. RT. Reaction time; MT. Movement time; PV. Peak velocity; HDT. Head-down tilt; R. Recovery.

Fig. 6 shows the temporal trends in cognitive-motor task indicators across the experimental phases, with significant post-hoc pairwise comparison results. Detailed statistical analysis and indicator values are provided in Additional file 1**:** Table S4. RT (beep) showed differences (*χ*²=20.36, *P*=0.001), with post-hoc analysis confirming significant pairwise effects at multiple time points. RT (no-beep) demonstrated even stronger differences (*χ*²=56.20, *P*<0.001), supported by robust post-hoc results. In contrast, MT (beep) showed only marginal variation (*χ*²=13.07, *P*=0.023), which did not reach significance in post-hoc comparisons. MT (no-beep) exhibited marked differences (*χ*²=49.54, *P*<0.001), confirmed by post-hoc tests. PV (beep) remained stable throughout (*χ*²=1.45, *P*=0.919), whereas PV (no-beep) showed significant alterations (*χ*²=44.84, *P*<0.001) with consistent post-hoc confirmation.

### Correlations between total cerebral blood inflow changes and cognitive-motor performance

**Fig. 7:**
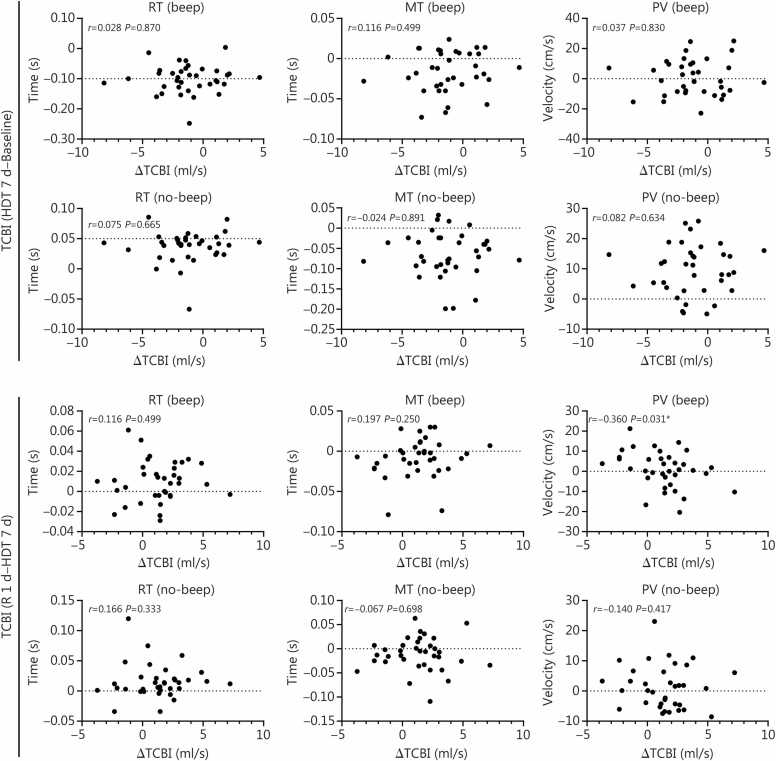
**Associations between changes in TCBI and cognitive-motor performance across two intervals**. Top row: change in TCBI from baseline to HDT 7 d. Bottom row: change in TCBI from HDT 7 d to R 1 d. Each panel shows individual participants. Spearman’s correlation coefficient (*r*) and *P-*value are annotated. TCBI. Total cerebral blood inflow; RT. Reaction time; MT. Movement time; PV. Peak velocity; HDT. Head-down tilt; R. Recovery.

As shown in Fig. 7, correlation analyses were conducted to examine the relationship between changes in TCBI and cognitive-motor performance measures. When comparing TCBI at HDT 7 d with baseline, no significant correlations were observed with RT, MT, or PV, either with or without auditory cues (all *P-*values>0.05). In contrast, when comparing TCBI at R 1 d with HDT 7 d, most measures again showed no significant associations, except for PV with auditory cues, which demonstrated a significant negative correlation with TCBI changes (Spearman *r*=−0.360, *P*=0.031).

## Discussion

In this study, we employed 4D flow MRI to assess changes in average blood flow and vascular morphology of major intracranial arteries during a 7-day −6° HDT period and a subsequent 5-day recovery phase. Analysis of variance and post-hoc testing revealed no significant changes in vessel lumen area across the major intracranial arteries. However, adaptive patterns of average flow differed across arteries, with prominent anterior-circulation changes in which the bilateral MCAs showed significant changes with broadly parallel trajectories, whereas the bilateral ICAs exhibited significantly heterogeneous adaptation patterns, and posterior-circulation flow remained relatively stable. In parallel, among systemic hemodynamic parameters, only SBP was identified as a significant predictor of the reduction in cerebral inflow from baseline to HDT 7 d, suggesting that systemic pressure regulation plays a key role in modulating cerebral perfusion under simulated microgravity. Endocrine analysis suggested that higher baseline cortisol levels might be linked to greater reductions in cerebral blood inflow during HDT, highlighting the potential involvement of stress-related hormonal regulation. Moreover, cognitive-motor performance exhibited significant phase-related variations, indicating adaptive but reversible functional changes. However, the relationship between cerebral inflow dynamics and behavioral outcomes appeared weak, suggesting that short-term hemodynamic adaptations may not directly translate into measurable cognitive effects under simulated microgravity.

We found heterogeneous temporal adaptations of arterial flow during HDT and recovery. BA showed relatively minor fluctuations without statistical significance, suggesting low sensitivity to headward fluid shifts (Fig. 3**a**). By contrast, the ICAs exhibited more pronounced modulation, with ICAL exhibiting an early compensatory rise at HDT 12 h, followed by a significant decline at HDT 3 d and HDT 7 d, and then a rebound exceeding baseline during early recovery (Fig. 3**b**). ICAR displayed a progressive reduction beginning at HDT 12 h, reaching a nadir at HDT 3 d and showing a comparatively blunted recovery relative to the contralateral side (Fig. 3**c**). Consistent with the paired-artery analysis, both the ICAs and MCAs showed significant time main effects, indicating overall temporal changes in bilateral average flow during HDT and recovery. However, no significant side main effect was detected, suggesting that there was no persistent overall left-right difference across phases. Notably, only the ICAs showed a significant time×side interaction, indicating phase-dependent lateralized divergence, with ICAL exceeding ICAR at HDT 12 h and during recovery phase (Table 3**;** Fig. 4**a**). One plausible interpretation is that the left-sided inflow may readjust more rapidly during posture transitions, given its closer proximity to the heart and potentially earlier exposure to systemic hemodynamic changes. Both MCAs declined markedly during HDT, with the steepest reductions at HDT 3 d. MCAL remained persistently lower than baseline despite partial recovery, whereas MCAR recovered rapidly and exceeded baseline during recovery (Fig. 3**d,e**). By contrast, although the MCAs also exhibited significant temporal variation overall, they did not show a significant time×side interaction, suggesting broadly parallel bilateral adaptation patterns rather than phase-dependent lateralized divergence (Table 3**;** Fig. 4**b**). This pattern may be related to collateral pathways within the CoW, which can redistribute inflow between the paired ICAs and thereby buffer hemispheric disparities at more distal branches, potentially attenuating distal bilateral differences despite upstream ICAs asymmetry. In line with this interpretation, the $S_{\mathrm{CoW}}$ was higher on the left (left vs. right: 3.8±1.3 vs. 3.2±1.0, *P*=0.009), indicating modest hemispheric asymmetry in anatomic collateral potential that may facilitate side-dependent inflow partitioning while maintaining distal flow balance. Collectively, these findings indicate that anterior circulation vessels, particularly MCAs [41], are more sensitive to posture-induced hemodynamic alterations than BA and suggest that considering artery-specific bilateral differences may be relevant when optimizing measures to restore cerebral perfusion during spaceflight analogs [42]. Accordingly, countermeasure strategies may benefit from accounting for left-right heterogeneity in paired-artery responses rather than assuming uniform hemispheric adaptations.

Our findings reveal that the regulation of cerebral blood flow (CBF) during recovery occurs more rapidly than the adaptive decline observed during the HDT phase. Specifically, TCBI demonstrated a gradual decline over several days during HDT, reaching a statistically significant reduction at HDT 7 d compared to both baseline (*P*=0.035) and HDT 12 h (*P*=0.012). In contrast, upon cessation of HDT, a significant rebound in TCBI was observed as early as R 1 d (*P*=0.025), indicating a prompt cerebrovascular response to the restoration of upright posture. Furthermore, binary logistic regression analysis revealed that SBP was the only significant predictor of a >10% reduction in TCBI during the HDT phase (Table 4, *OR*=3.004, 95% CI 1.028−8.777), suggesting that elevated systolic pressure may play a critical compensatory role in cerebral perfusion regulation under simulated microgravity. The logistic regression analysis for the recovery phase (Table 5) revealed no significant associations between the increase in TCBI and systemic hemodynamic variables, including HR, SBP, DBP, and MAP. This lack of significant predictors may be attributed to the rapid rebound of TCBI following the resumption of upright posture, suggesting that cerebral perfusion regulation during recovery is swift and may rely less on systemic hemodynamic factors such as blood pressure.

These findings have important implications for astronaut health and the development of countermeasures for spaceflight-induced cerebrovascular changes [18]. Prolonged reductions in TCBI and asymmetric arterial responses may contribute to impaired cerebral perfusion and increased risk of neurocognitive deficits in microgravity-exposed individuals [43], [44]. Understanding these adaptations is essential for designing targeted interventions, such as artificial gravity, fluid loading strategies, or pharmacological approaches to maintain cerebral perfusion in astronauts [45]. Furthermore, these findings have clinical relevance for bedridden patients, where prolonged supine positioning may induce similar cerebrovascular changes, emphasizing the need for preventive strategies in long-term bed rest conditions [1], [44].

Prior studies have examined cerebral hemodynamics during HDT, but most were limited to single time points or isolated vessels [1], [46], [47]. Kato *et al.* [48] employed transcranial Doppler to assess MCA velocity during a brief 10-minute exposure to −10° or −30° HDT and reported no significant differences [baseline: (64 ±13) cm/s; −10°: (65±9) cm/s; −30°: (63±10) cm/s; *P*=0.467], despite an observed increase in optic nerve sheath diameter suggestive of elevated intracranial pressure (ICP). In contrast, our 7-day HDT protocol revealed that the MCAs exhibited the highest temporal sensitivity among all major intracranial arteries, with more significant changes detected across multiple time points. These findings suggest that longer durations of simulated microgravity may reveal dynamic cerebrovascular adaptations that remain undetectable in short-term protocols.

The progressive decline in TCBI during HDT and its rapid increase during recovery align with the expected hemodynamic responses to cephalad fluid shifts in simulated microgravity. Seiller *et al.* [20] using arterial spin labeling MRI, reported a mean CBF reduction of 5.8% following −15° HDT in healthy individuals. Although our study measured TCBI rather than tissue-level CBF, both parameters reflect global cerebral perfusion [34]. Therefore, our findings are consistent with arterial spin labeling-based studies demonstrating decreased cerebral perfusion during HDT. In addition, previous studies have suggested that an initial elevation in ICP during HDT may trigger cerebrovascular autoregulatory vasoconstriction to preserve cerebral perfusion pressure, ultimately leading to reduced cerebral arterial inflow [18], [48], [49]. This physiological adaptation may account for the observed gradual decline in TCBI during the HDT phase. Notably, few studies have reported on CBF changes during the recovery phase [7], [50]. Our findings show that TCBI rebounded rapidly at R 1 d, with recovery occurring faster than the preceding decline during HDT. This asymmetric adaptation highlights a novel aspect of cerebral perfusion regulation and emphasizes the importance of evaluating the recovery phase when assessing the hemodynamic effects of simulated microgravity.

The significant association between SBP and TCBI observed during the HDT phase indicates a potential link between systemic blood pressure and cerebral perfusion regulation under conditions of fluid redistribution. An increase in SBP may reflect a compensatory response to headward fluid shifts, helping to maintain cerebral perfusion pressure and thus sustain cerebral inflow. As reported by Lee *et al.* [51], HDT may lead to enhanced sympathetic nervous system activity and altered baroreflex sensitivity, which could contribute to blood pressure-mediated cerebrovascular adjustments. Moreover, autoregulatory mechanisms in the cerebral vasculature may differ in their response to systolic vs. diastolic pressure. Webb *et al.* [52] have shown that the systolic component of blood pressure, namely SBP and PP, has a greater influence on cerebral flow than diastolic pressure and may exert this effect independently of MAP. These findings lend physiological plausibility to our observation that SBP elevation is significantly associated with reductions in TCBI during HDT.

The finding that higher baseline cortisol predicted a ≥10% reduction in TCBI from baseline to HDT 7 d suggests a role for hypothalamic-pituitary-adrenal (HPA) axis tone in shaping macrovascular inflow during posture-induced fluid shifts. Cortisol can impair endothelial function, reduce nitric oxide bioavailability, and augment vasoconstrictor responsiveness, thereby increasing cerebrovascular resistance and narrowing the vasodilatory reserve available to buffer systemic hemodynamic perturbations [53]. Individuals with higher basal cortisol may therefore be more susceptible to flow reductions when HDT alters central blood volume and baroreflex loading. By contrast, baseline renin was not a significant predictor, which aligns with the physiology that renin-angiotensin-aldosterone system (RAAS) activity is strongly posture- and volume-dependent [54]. A single supine baseline measurement may not capture the dynamic RAAS adaptations elicited by HDT. These findings may suggest the hypothesis that HPA-linked vascular reactivity, rather than resting RAAS tone, contributes to inter-individual variability in the early decline of intracranial arterial inflow during HDT [55].

Across experimental phases, RT and MT declined, with larger and more consistent reductions in the no-beep condition. PV remained stable in the beep condition but increased in the no-beep condition. These time courses suggest that when movements are generated without external cueing, preparation becomes faster and execution shorter, yet movement vigor diminishes [38]. During the HDT phase, the decrease in TCBI did not correlate with any cognitive-motor measure, suggesting no clear inflow-behavior association within the HDT condition. During early recovery, only one exploratory exception was observed between HDT 7 d and R 1 d, where TCBI recovery showed a modest negative correlation with PV in the beep condition (*r*=−0.360, *P*=0.031). Given the exploratory nature of this analysis and the isolated significant association observed, this finding should be interpreted as a preliminary signal rather than evidence of direct vascular behavior coupling. Accordingly, the pattern may reflect strategic adjustments during the recovery posture transition rather than a perfusion-driven causal mechanism. Participants with greater inflow rebound exhibited lower movement vigor in cued trials at R 1 d. A plausible interpretation is partial decoupling between macrovascular inflow and the control of movement vigor, reflecting strategic adjustments under phasic alerting rather than a direct perfusion-behavior coupling [56]. Notably, Mekari *et al.* [57] have also suggested that hemodynamic changes during acute −6° HDT do not necessarily result in a proportional improvement in cognitive performance. This is consistent with the lack of robust inflow-behavior coupling observed here. In view of the posture change from HDT to recovery, these cognitive-motor findings should be considered exploratory, and future studies should include a posture-matched baseline or standardize posture across sessions to more cleanly separate microgravity-analog effects from posture effects. Overall results indicate that compared to medium-to-long-term HDT [19], we did not observe evidence of a marked cognitive decline over 7-day HDT.

Despite these novel findings, this study has some limitations. First, the sample size was relatively small and included only male participants aged 18−58 years, which may limit the generalizability of the findings. Given the influence of sex hormones on cerebrovascular regulation, extrapolation to women should be made with caution. Future studies should include female participants to improve external validity. Second, the study lacked a comparison resting-state or task-based baseline condition, making it difficult to disentangle global hemodynamic adaptations from task-related changes in CBF. Meanwhile, future research will aim to investigate multiple HDT angles to assess the dose-response relationship between the degree of tilt and cerebrovascular adaptation, which may provide insight into potential thresholds or nonlinear responses under simulated microgravity. Third, while HDT is widely employed to model microgravity effects, it does not fully replicate the full spectrum of spaceflight-related physiological changes, such as alterations in brain metabolism, cerebrospinal fluid dynamics, and metabolic profiles [10], [22]. Future studies should integrate additional measurements to strengthen mechanistic understanding.

## Conclusions

This study delineates a vessel-specific and lateralized pattern of cerebral arterial flow adaptation during a 7-day −6° HDT and a 5-day recovery period, indicating that anterior-circulation inflow is more responsive than posterior-circulation inflow to posture-induced fluid shifts. The system-level trajectory was characterized by a gradual reduction in TCBI during HDT, followed by a rapid rebound after re-ambulation in the recovery phase. Interindividual variability in TCBI reduction was linked to systemic regulation, with blood pressure control and baseline endocrine status emerging as key correlates of individual susceptibility. In contrast, phase-dependent cognitive-motor changes demonstrated limited coupling with macrovascular inflow dynamics, implying that short-term behavioral adaptation may proceed independently of TCBI fluctuations. Together, these findings emphasize the importance of targeting the early recovery window and provide a mechanistic rationale for developing countermeasures that stabilize systemic blood pressure and mitigate endocrine stress to preserve cerebrovascular resilience in spaceflight.

## Abbreviations

4D flow MRI: Four-dimensional flow magnetic resonance imaging
CaBF: Cerebral arterial blood flow
CBF: Cerebral blood flow
CIs: Confidence intervals
CoW: Circle of Willis
DBP: Diastolic blood pressure
HDT: Head-down tilt
HPA: Hypothalamic-pituitary-adrenal
HR: Heart rate
ICAL: Left internal carotid artery
ICAR: Right internal carotid artery
ICAs: Internal carotid arteries
ICC: Intraclass correlation coefficient
ICP: Intracranial pressure
MAP: Mean arterial pressure
MCAL: Left middle cerebral artery
MCAR: Right middle cerebral artery
MCAs: Middle cerebral arteries
MT: Movement time
*OR*: Odds ratio
PP: Pulse pressure
PV: Peak velocity
R: Recovery
RAAS: Renin-angiotensin-aldosterone system
RM ANOVA: Repeated-measures analysis of variance
RT: Reaction time
SBP: Systolic blood pressure
SpO_2_: Oxygen saturation
TCBI: Total cerebral blood inflow
TOF-MRA: Time-of-flight magnetic resonance angiography

## Funding

This work was supported by the Space Medical Experiment Project of China Manned Space Program (HYZHXMH01005), the Beijing Hospitals Authority Innovation Studio of Young Staff Funding Support (202302), and Beijing Scholar 2015.

## Data Availability

The data of this study are available from the corresponding authors upon request.
