# Supplementary material for: Lateralized cerebral arterial blood flow and blood pressure adaptations to short-term head-down tilt: a 4D flow MRI study with cognitive function assessment
Source: Mil Med Res. 2026 Jun 23;13(1):100048. doi: 10.1016/j.mmr.2026.100048 (PMC13315500; doi:10.1016/j.mmr.2026.100048)
Supplement: Supplementary file 1 — Supplementary material [file mmc1.pdf]

Materials and methods

Quality control protocol for biochemical assays

Blood specimens were analyzed in the hospital's accredited central laboratory according to the manufacturers' instructions for the chemiluminescent immunoassays. Pre-analytical procedures were standardized with overnight fasting, fixed sampling time points, and routine sample handling/storage procedures. Internal quality control materials at low and high concentration levels were run routinely during assay use, including after calibration and when a new reagent lot was introduced. Results were accepted only when quality control values fell within the predefined laboratory ranges; otherwise, the run was repeated and corrective procedures were undertaken according to laboratory policy. When available, duplicate measurements were averaged. Laboratory personnel were blinded to imaging outcomes.

Detailed methods for cerebral arterial blood flow analysis

Before data processing, regions of interest (ROIs) were manually selected on the coronal images to delineate the major intracranial arteries. During preprocessing, static tissue and vessel masks were automatically generated, and offset correction (OC) was applied using static-tissue referencing to mitigate background phase offsets and reduce phase noise. Phase anti-aliasing was applied in cases where phase aliasing artifacts were detected. Vessel segmentation was performed using cardiovascular imaging (CVI) 42's (Circle Cardiovascular Imaging Inc., Calgary, Canada) automatic lumen contouring, followed by manual supervision. Specifically, the automatic contouring applies intensity-based thresholding on magnitude images with region-growing and temporal smoothing across the cardiac phases to ensure contour consistency. When necessary, operators manually adjusted local

thresholds and seed points to ensure that the lumen-wall boundaries accurately aligned with both magnitude and phase images. Centerline extraction and mask-threshold adjustment were employed to refine the segmentation and ensure accurate delineation of vessel walls. For flow quantification, analysis planes were positioned perpendicular to the vessels, and blood flow parameters were calculated across the cardiac cycle. All ROIs and flow curves were visually inspected to ensure quality and accuracy, with manual corrections applied as needed. For quality control, planes were required to: 1) be orthogonal to the local centerline, 2) lie within a visually straight segment with no side branches within 3 mm, and 3) remain at least one local vessel diameter from the bifurcation.

Cognitive-motor task procedures and preprocessing

Each session comprised 120 pseudorandomized trials. After the right index finger was placed on a start circle, a target appeared following a 500–1000 ms variable period at one of three directions (45°, 90°, and 135°) located 12 cm from the start. In half of the trials, a 50 ms auditory cue (beep) was presented at target onset to modulate action preparation; reaction time (RT, target onset to movement onset), movement time (MT, movement onset to movement offset), and peak velocity (PV, maximum speed during the movement) were recorded separately for the beep and no-beep conditions. Participants were instructed to move as quickly and accurately as possible, pause briefly at the target, and then return to the start. Trials in which the interval from target onset to movement end exceeded 650 ms were classified as invalid.

Table S1 Inter-rater reliability for vessel-specific flow measurements (*n*=40)

Vessel	ICC (A, 1)	95% CI
BA	0.93	(0.79−0.98)
ICAL	0.97	(0.51−0.99)
ICAR	0.93	(0.76−0.99)
MCAL	0.98	(0.93−0.99)
MCAR	0.97	(0.81−0.99)

Inter-rater reliability was assessed using intraclass correlation coefficients (ICC) for vessel-specific flow measurements in the basilar artery (BA), left and right internal carotid arteries (ICAL/ICAR), and left and right middle cerebral arteries (MCAL/MCAR). From the full dataset (304 4D Flow MRI scans; 38 participants × 8 time points), 40 scans were randomly selected using a time-point-stratified sampling strategy (5 scans per time point). Two independent raters with prior CVI42 annotation experience independently performed vessel segmentation/ROI placement and extracted CVI-derived metrics following the standardized plane placement and contouring criteria and quality-control rules described in the Methods. ICCs are reported as ICC (A, 1) (two-way mixed-effects model, absolute agreement, single measurement) with 95% confidence intervals (CIs). ROI. Region of interest; CVI. cardiovascular imaging

Table S2 Standardized Circle of Willis (CoW) collateral capacity score: measurement definitions and scoring thresholds (0−9 per hemisphere)

Score	Collateral route	Measurements (mm)	Measurement definition and location (based on TOF-MRA)	Normalized ratio used for scoring	0−3 point scoring thresholds*	Handling absence/non-visualization
S_{ACom}	Anterior cross-filling via the ACom complex	D_{MCA}, $D_{\mathrm{A1,contra}}$, D_{ACom}	D_{MCA}: ipsilateral proximal MCA M1 diameter, measured on a straight segment pre-bifurcation;<br>$D_{\mathrm{A1,contra}}$: contralateral ACA A1 diameter (the A1 supplying cross-filling toward the indexed hemisphere), measured on a straight segment;<br>D_{ACom}: narrowest luminal diameter of the ACom;<br>Effective anterior diameter: $D_{\mathrm{forward}}=\min(D_{\mathrm{A1,contra}}, D_{\mathrm{ACom}})$	$R_{\mathrm{forward}}=\frac{D_{\mathrm{forward}}}{D_{MCA}}=\frac{\min(D_{\mathrm{A1,contra}},D_{\mathrm{ACom}})}{D_{\mathrm{MCA}}}$	3: $R_{\mathrm{forward}}\geq 0.8$;<br>2: $0.5\leq R_{\mathrm{forward}}<0.8$;<br>1: $0.2\leq R_{\mathrm{forward}}<0.5$;<br>0: $R_{\mathrm{forward}}<0.2$	If ACom or contralateral A1 is absent/non-visualized to set the corresponding diameter to 0, resulting in $S_{\mathrm{ACom}}=0$
S_{PCom}	Posterior collateral via ipsilateral PCom	D_{PCom}, D_{MCA}	D_{PCom}: narrowest luminal diameter of the ipsilateral PCom;<br>D_{MCA}: as defined above	$R_{\mathrm{PCom}}=\frac{D_{\mathrm{PCom}}}{D_{\mathrm{MCA}}}$	3: $R_{\mathrm{PCom}}\geq 0.8$;<br>2: $0.5\leq R_{\mathrm{PCom}}<0.8$;<br>1: $0.2\leq R_{\mathrm{PCom}}<0.5$;<br>0: $R_{\mathrm{PCom}}<0.2$	If PCom is absent/non-visualized to set $D_{\mathrm{PCom}}=0$, resulting in $S_{\mathrm{PCom}}=0$
S_{P1}	Indirect posterior route indexed by ipsilateral PCA P1 caliber	D_{P1}, D_{MCA}	D_{P1}: ipsilateral PCA P1 diameter, measured on a straight proximal P1 segment;<br>D_{MCA}: as defined above	$R_{\mathrm{P1}}=\frac{D_{\mathrm{P1}}}{D_{\mathrm{MCA}}}$	3: $R_{\mathrm{P1}}\geq 1.0$;<br>2: $0.7\leq R_{\mathrm{P1}}<1.0$;<br>1: $0.4\leq R_{\mathrm{P1}}<0.7$;<br>0: $R_{\mathrm{P1}}<0.4$	If P1 is absent/non-visualized to set $D_{\mathrm{P1}}=0$, resulting in $S_{\mathrm{P1}}=0$

Score	Collateral route	Measurements (mm)	Measurement definition and location (based on TOF-MRA)	Normalized ratio used for scoring	0−3 point scoring thresholds[*]	Handling absence/non-visualization
S_{CoW}	Total CoW collateral capacity score	S_{ACom}, S_{PCom}, S_{P1}	Computed separately for each hemisphere	$S_{CoW}=S_{ACom}+S_{PCom}+S_{P1}$	Range: 0−9 (sum of three 0−3 component scores)	Hemisphere scoring requires measurable D_{MCA}; Otherwise, the hemisphere is excluded from scoring

[*]Thresholds are applied to the normalized ratios defined in the table, and each component score takes an integer value from 0 to 3. MCA. Middle cerebral artery; M1. M1 segment of the MCA; ICA. Internal carotid artery; ACA. Anterior cerebral artery; A1. A1 segment of the ACA; ACom. Anterior communicating artery; PCA. Posterior cerebral artery; P1. P1 segment of the PCA; PCom. Posterior communicating artery; BA. Basilar artery; Ipsilateral. Same side as the indexed hemisphere; Contralateral. Opposite side to the indexed hemisphere; TOF-MRA. Time-of-flight magnetic resonance angiography

Table S3 Statistical analysis of blood flow (ml/s) changes in intracranial major arteries across different time points during HDT and recovery phases (*n*=38)

Artery	Time points								RM ANOVA		Friedman test			Post-hoc significance
	Baseline	HDT 12 h	HDT 1 d	HDT 3 d	HDT 7 d	R 1 d	R 3 d	R 5 d	*F*	*P*-value	*df*	χ^2	*P*-value	
BA[a] (mean±SD)	3.3±0.96	3.4±0.95	3.5±0.89	3.2±0.72	3.1±0.91	3.3±0.82	3.4±0.99	3.3±1.1	0.787	0.599	-	-	-	NA
ICAL[b] [median (IQR)]	5.2 (4.9–5.9)	5.2 (4.6–6.6)	5.0 (4.5–6.2)	4.7 (4.2–5.3)	4.7 (4.2–5.5)	5.4 (4.5–6.5)	5.4 (4.5–6.3)	5.3 (4.6–6.1)	-	-	7	34.16	<0.001	Yes
ICAR[b] [median (IQR)]	5.3 (4.4–6.1)	5.0 (4.6–6.0)	4.9 (4.3–5.4)	4.7 (3.7–5.3)	4.6 (4.0–5.3)	4.8 (4.1–5.8)	4.8 (4.3–5.4)	4.6 (4.0–5.4)	-	-	7	16.35	0.022	No
MCAL[b] [median (IQR)]	3.7 (3.2–3.9)	3.7 (3.2–4.3)	3.5 (3.1–3.9)	3.1 (2.8–3.4)	3.0 (2.7–3.6)	3.5 (3.2–3.8)	3.5 (3.2–4.0)	3.5 (3.2–3.8)	-	-	7	73.11	<0.001	Yes
MCAR[b] [median (IQR)]	3.5 (3.1–3.9)	3.7 (3.4–4.0)	3.5 (2.9–3.8)	3.2 (2.8–3.6)	3.1 (2.8–3.6)	3.6 (3.2–3.9)	3.6 (3.3–3.8)	3.5 (3.0–4.0)	-	-	7	49.02	<0.001	Yes

[a]Repeated-measures analysis of variance (RM ANOVA); [b]The Friedman test. "-". No data. BA. Basilar artery; ICAL. Left internal carotid artery; ICAR. Right internal carotid artery; MCAL. Left middle cerebral artery; MCAR. Right middle cerebral artery; HDT. Head-down tilt; R. Recovery; NA. Not applicable

Table S4 Statistical analysis of cognitive-motor outcome changes across different time points during HDT and recovery phases (*n*=36) [median (IQR)]

Variable	Time points						Friedman test			Post-hoc significance
	Baseline	HDT 1 d	HDT 3 d	HDT 7 d	R 1 d	R 5 d	*df*	χ^2	*P*-value	
RT (beep) (s)	0.28 (0.27–0.30)	0.29 (0.27–0.30)	0.28 (0.27–0.30)	0.28 (0.27–0.30)	0.28 (0.26–0.29)	0.27 (0.26–0.29)	7	20.36	0.001	Yes
RT (no-beep) (s)	0.32 (0.30–0.35)	0.31 (0.30–0.33)	0.30 (0.28–0.32)	0.30 (0.28–0.30)	0.28 (0.27–0.30)	0.29 (0.27–0.31)	7	56.20	<0.001	Yes
MT (beep) (s)	0.27 (0.26–0.30)	0.26 (0.25–0.28)	0.26 (0.24–0.29)	0.26 (0.24–0.28)	0.26 (0.25–0.29)	0.26 (0.25–0.28)	7	13.07	0.023	No
MT (no-beep) (s)	0.43 (0.40–0.46)	0.36 (0.35–0.41)	0.37 (0.34–0.40)	0.37 (0.32–0.40)	0.39 (0.34–0.42)	0.37 (0.31–0.43)	7	49.54	<0.001	Yes
PV (beep) (cm/s)	79 (69–85)	81 (72–86)	80 (71–90)	82 (71–90)	81 (69–88)	82 (70–87)	7	1.45	0.919	NA
PV (no-beep) (cm/s)	43 (39–48)	51 (46–56)	52 (47–60)	51 (45–63)	48 (44–58)	52 (42–67)	7	44.84	<0.001	Yes

"Beep" indicates trials performed with an auditory cue, and "no-beep" indicates trials performed without the auditory cue. RT. Reaction time; MT. Movement time; PV. Peak velocity; NA. Not applicable; HDT. Head-down tilt
